# The Attention Habit II: How selection history shapes the strategic control of attention

**DOI:** 10.3758/s13423-025-02790-7

**Published:** 2025-12-10

**Authors:** Brian A. Anderson, David S. Lee, Niya Yan, Molly R. McKinney, Andrew Clement

**Affiliations:** 1 Department of Psychological & Brain Sciences, Texas A&M University, 4235 TAMU, College Station, TX 77843-4235 USA; 2 Millsaps College, Jackson, MS USA

**Keywords:** Attentional control, Selection history, Habit learning, Motivation, Intention, Goals

## Abstract

The allocation of attention is now widely understood to reflect the joint influence of goal-directed, stimulus-driven, and selection history-driven mechanisms of control. The influence of selection history is often discussed in the context of the involuntary control of attention, competing with goal-directed influences. Here, we argue that selection history also influences voluntary, goal-directed mechanisms of control, shaping the manner in which intentional prioritization of stimuli occurs. In this respect, the habitual guidance of attention is not limited to mechanisms of priority assignment that operate without respect to observers’ goals and intentions; rather, the goal-directed control of attention itself is sensitive to habit-like mechanisms of priority assignment. This has implications for how we characterize mechanisms of attentional control, blurring the distinction between goal-directed and selection history-driven influences and raising important questions concerning the degree to which the intentional control of attention is biased by prior learning.

## Voluntary and involuntary mechanisms of attentional control

Attention is the mechanism by which the brain selectively processes a subset of perceptual input, allowing important information to be prioritized at capacity-limited stages of information processing (Desimone & Duncan, 1995). To survive and thrive, the information most critical to the guidance of efficacious, contextually appropriate behavior must be selectively attended (e.g., Anderson, 2013, 2021a). Research on the control of attention has long been interested in understanding the mechanisms by which the attention system prioritizes some stimuli in the environment over others.

One of the oldest distinctions in theories of attentional control concerns the contrast between voluntary and involuntary mechanisms of selection (e.g., Jonides, 1981; Näätänen et al., 1980). On one hand, what observers prioritize via attention can be influenced endogenously by representations linked to task instruction. Observers can prioritize stimuli possessing a particular feature that is characteristic of the target they are looking for (e.g., Anderson, 2014; Wolfe, 2021; Wolfe et al., 1989), or they can prioritize stimuli presented in a particular region of visual space (e.g., Posner, 1980; Yantis & Johnston, 1990), allocating their attention in accordance with information provided to them in advance of the stimulus array. A hallmark of goal-directed attention is that it can be flexibly applied, allowing for online adjustments in response to changing task demands (see Theeuwes, 2018).

On the other hand, the allocation of attention can sometimes proceed in a manner contrary to observers’ task goals. For example, physically salient stimuli (i.e., stimuli with high local feature contrast; see Itti & Koch, 2001) are sometimes preferentially attended even when they are known to be irrelevant to the task at hand (e.g., Luck et al., 2021; Theeuwes, 1992, 2010; Yantis & Jonides, 1984). Similarly, while attentional control settings can be established voluntarily, the influence of such control settings can overgeneralize, resulting in the involuntary selection of stimuli that possess a target-defining feature that participants nonetheless know to be task-irrelevant (e.g., Folk & Anderson, 2010; Folk et al., 1992). When attention is allocated to a non-target stimulus involuntarily despite the intention to allocate attention elsewhere, attention can be said to have been *captured* by the eliciting stimulus (Liesefeld et al., 2024). Although the extent to which attentional capture by physically salient stimuli can be suppressed or otherwise prevented by attentional control settings is debated (see, e.g., Anderson & Folk, 2012; Gaspelin & Luck, 2018; Luck et al., 2021), it is widely accepted that physically salient stimuli can, under certain task conditions, elicit the capture of attention. In many cases, attentional priority reflects a combination of voluntary, goal-directed and involuntary, stimulus-driven influences (see, e.g., Bacon & Egeth, 1994; Folk et al., 1992; Luck et al., 2021; Serences et al., 2005).

Consistent with a distinction between voluntary and involuntary attentional control, an anatomically dissociable pair of neural networks—the dorsal and ventral attention networks— have been linked to goal-directed and stimulus-driven attention allocation, respectively (e.g., Corbetta et al., 2008; Corbetta & Shulman, 2002; Vossel et al., 2014). There appears to be one neural circuit (i.e., the *dorsal attention network*) that supports maintaining and applying current attentional control settings, and another network (i.e., the *ventral attention network*) that can act as an attentional “circuit breaker,” interrupting goal-directed attentional control when unexpected or otherwise salient stimuli are encountered (Corbetta et al., 2008; Corbetta & Shulman, 2002). This circuit breaker function of involuntary attentional control is thought to help ensure that potentially pertinent stimuli receive consideration and that current attentional control settings can be reevaluated and potentially updated in a dynamic environment.

## Selection history as an involuntary mechanism of control

Beyond the influence of goals and physical salience, the allocation of attention is now widely understood to be subject to a third distinct mechanism of attentional control driven by prior experience or *selection history* (Anderson et al., 2021; Awh et al., 2012). The experiences observers have had with different stimuli and the manner in which they have selectively processed these stimuli in the past can influence how attention is allocated to stimuli in the future. For example, stimuli previously associated with reward or punishment are preferentially attended (e.g., Anderson et al., 2011; Anderson & Britton, 2020; Anderson & Halpern, 2017; Anderson & Kim, 2019; Le Pelley et al., 2015; Schmidt et al., 2015), as are stimuli that consistently served as a target of visual search (e.g., Anderson & Britton, 2019; H. Kim & Anderson, 2019b; Kyllingsbaek et al., 2001; Sha & Jiang, 2016; Shiffrin & Schneider, 1977). Locations that have frequently contained a target will be prioritized in visual search (e.g., Geng & Behrmann, 2002, 2005; Jiang et al., 2013; Jiang & Swallow, 2013; Won & Leber, 2016), and locations that have frequently contained a distractor will be deprioritized (e.g., Britton & Anderson, 2020; Goschy et al., 2014; A. J. Kim & Anderson, 2022; B. Wang & Theeuwes, 2018a, 2018b, 2018c; for an in-depth discussion of the variety of selection history-dependent influences on the control of attention, see Anderson et al., 2021). Such learning-dependent influences on the control of attention can be robustly observed for stimuli that are physically non-salient, and these influences cannot be reduced to a modulatory influence of learning on the goal-directed control of attention (Anderson, 2013, 2016).

Anderson et al. (2011) demonstrated that previously reward-associated stimuli could involuntarily capture attention even when they were physically non-salient, known to be task-irrelevant, and no longer associated with reward (and indeed rewards were no longer available in the task). This finding provided strong evidence for learning history having a direct influence on the control of attention that could compete with goal-directed influences and the influence of physical salience. This striking result helped to usher in an intense research focus on the involuntary nature of selection history-dependent influences on the control of attention, research that has produced a variety of results convincingly showing that the control of attention is shaped by learning in ways over which observers have limited control (e.g., Anderson, 2021c; Anderson & Britton, 2020; Grégoire et al., 2022; H. Kim & Anderson, 2019a, 2021; Le Pelley et al., 2015). Such findings are consistent with the idea that the control of attention can proceed in a habit-like manner, with habitual attention contrasting with goal-directed influences (see Anderson, 2016; Hikosaka et al., 2013; Jiang, 2018; H. F. Kim et al., 2015).

## Models of habitual attention

Focusing largely on reward-related attentional biases, Anderson (2016) argued that the allocation of attention can proceed as a habit-like response to a valued stimulus (see also Hikosaka et al., 2013), a position expanded upon by Jiang (2018) in the context of target location probability cuing. One of the hallmarks of habit-like attention that distinguishes it from goal-directed attention is its characteristic inflexibility; habit-like attention can be strikingly robust to changes in the contingencies of the environment (e.g., Anderson et al., 2012; Jiang & Swallow, 2013; Mine & Saiki, 2015) and habit-like attentional biases can be markedly slow to update in the face of contingencies that no longer favor the bias (e.g., Anderson, 2016; Anderson et al., 2011; Anderson & Yantis, 2013; Jiang, 2018; Liao & Anderson, 2020a). Habit-like attentional biases can also operate implicitly, without conscious awareness or direction (Anderson et al., 2021; Jiang, 2018).

Habit-like attention, and the influence of selection history on the control of attention more broadly, has consistently been characterized as distinct from goal-directed (as well as stimulus-driven) attention, supported by dissociable mechanisms and capable of competing with goal-directed influences (e.g., Anderson, 2016; Anderson et al., 2021; Awh et al., 2012; Jiang, 2018). Although commonalities between voluntary, goal-directed mechanisms and selection history-driven mechanisms of attentional control have been discussed in a memory-driven framework for the control of attention (Anderson, 2024b), a clear distinction between the two remains, with very little in the way of theoretical bridging. The fact that selection history-dependent influences on the control of attention *can* involuntarily modulate attentional priority, however, should not be taken to imply that all selection history-dependent influences on the control of attention are involuntary in nature and operate outside of the purview of voluntary attentional control. In the present review paper, we propose a bridge that spans the gap between selection history and the voluntary control of attention.

Here, we argue that the voluntary control of attention is subject to selection history-driven influences, such that how a person chooses to search can be biased by prior learning in a habit-like manner. We argue for a second path by which selection history influences the control of attention: In addition to the established path by which selection history directly influences attentional priority computations (e.g., Anderson et al., 2021), there is an additional path by which selection history influences the processes involved in generating, maintaining, and applying the representations that are volitionally brought to bear in the guidance of attention. Through this second path, selection history manifests as a habit-like influence on how goal-directed attentional control proceeds, with selection history serving as a sort of “gravitational weight,” pulling the voluntary instantiation of an attentional control setting toward a particular state or configuration based on previous experience.

Before we explore evidence supporting our hypothesis, it is useful to define some key terms that contextualize our arguments. Although our hypothesis principally centers on the influence of selection history on *voluntary* attentional control—control mechanisms that are intentionally brought to bear in the assignment of attentional priority—our focus will be more specifically on instances of voluntary, *goal-directed* attentional control, as in such instances the goals of the task provide concrete boundary conditions for what the voluntary control of attention would be expected to look like. Attentional prioritization that serves in the interest of goal-attainment need not be voluntary in nature, as argued by Anderson (2018), and so our review of the literature places particular emphasis on studies that examine the *strategic control of attention* (see Irons & Leber, 2016, 2018), or the manner in which a person chooses what to prioritize when there are multiple viable attentional control settings by which task goals could be attained. Debates over the nature of free will notwithstanding (Dennet, 1984, 2004), the epitome of voluntary attentional control is a choice concerning how to search, and the strongest possible case for selection history influencing the voluntary control of attention can therefore be found in studies linking selection history to the attentional control settings a person chooses to adopt when searching.

When we discuss the influence of selection history on the voluntary control of attention as being *habit-like*, we refer to a *biasing influence* of selection history on attentional control settings. As characteristic of habit-like processes, this biasing influence is inflexible, able to proceed with limited awareness (i.e., not deliberate), and persists even when changes in the environment render it no longer beneficial. This is a non-traditional way of conceptualizing habit-like influences, which are often focused on behavioral output rather than internal representations. While the process of establishing and implementing an attentional control setting can be flexibly adjusted in accordance with momentary goals and motivations (e.g., Theeuwes, 2018; Wolfe, 2021), here we focus on antecedents of that process (i.e., experience-dependent factors that influence the choice of attentional control settings) and hypothesize these antecedents (i.e., selection history) serve as an additional source of input that can bias the end result (i.e., the attentional control setting that a person chooses to implement) in a habit-like manner.

## Linking selection history to the goal-directed control of attention

While considerable focus has been placed on how selection history is able to compete with goal-directed mechanisms of attentional control (e.g., Anderson, 2013; Anderson et al., 2021; Awh et al., 2012), more recent research has begun to explore how selection history might shape goal-directed attentional processes, potentially facilitating and/or biasing the manner in which observers intentionally prioritize sensory information. In this section, we highlight several studies that suggest an influence of selection history on voluntary, goal-directed attentional processes. In situations in which observers choose how to search and what to prioritize, we see evidence that selection history shapes the content of voluntarily instantiated attentional control states. That is, selection history shapes what a person chooses to search for and the manner in which they conduct their search. This is not to imply that goal-directed attentional processes are solely reducible to a product of selection history; rather, the assignment of attentional priority reflects a complex and dynamic weighting of stimulus input (see Anderson, 2021b; Narhi‐Martinez et al., 2023; Shomstein et al., 2023) governed by a variety of factors that vary in how flexibly and volitionally they can be adjusted. However, rather than reflecting two insular components of a trichotomy (see Awh et al., 2012), selection history and the voluntary, goal-directed control of attention possess meaningful overlap with respect to how they influence attentional priority computations.

### Attentional control settings as a function of reward and punishment history

Some of the earlier studies examining the modulatory influence of reward on attention included demonstrations that target processing could be facilitated when the target was currently or previously associated with reward (e.g., Della Libera & Chelazzi, 2009; Kiss et al., 2009; Raymond & O’Brien, 2009). Although such findings could be interpreted as reward history enhancing or otherwise sharpening goal-directed attentional processes, as reviewed in the preceding sections, it is also clear that reward history can have a more direct and involuntary influence on the control of attention (see Anderson, 2019) that could be at play here. The same can be said for the influence of stimulus‒punishment associations on the control of attention. As described by Anderson (2018, 2021a), involuntary influences of reward and punishment history likely serve in part to support the attentional processing of sought-after stimuli. Indeed, in many real-world scenarios, previously rewarded stimuli are also currently valued and intentionally prioritized, and likewise, observers choose to be vigilant for specific threats when they suspect that such threats might be present; in each of these cases, involuntary attentional biases toward such stimuli would complement goal-directed prioritization. Stronger evidence for an effect of reward and punishment history on voluntary, goal-directed attention per se would come from situations in which such history modulates the manner in which a person chooses to allocate attention when given a choice of how to search, especially when these choices are not influenced by current reward or punishment considerations and result in inefficient search.

The adaptive choice visual search (ACVS) task provides a situation in which observers are tasked with finding one of two color-defined targets from among stimuli that include non-targets rendered in the same two task-relevant colors. The distribution of non-target stimuli makes it such that searching among stimuli of one task-relevant color would be more efficient than searching from among stimuli of the other task-relevant color, which constitutes the “optimal” attentional strategy in the task (Irons & Leber, 2016, 2018; A. J. Kim et al., 2021a, 2021b). A recent study employing this task demonstrated that observers are biased to find and report a target that was rendered in a color previously associated with reward, even when searching in this manner was suboptimal (Lee et al., 2022). Conversely, observers were less likely to find and report a target that was rendered in a color previously associated with an aversive electric shock, even when they knew they could no longer be shocked and it was suboptimal to search in this way (Lee et al., 2025). Interestingly, highly anxious individuals showed the opposite tendency, suggesting that high anxiety might be associated with elevated threat monitoring (Lee et al., 2025), and by extension, that selection history may modulate goal-directed attention differently for different people.

In each of the above cases, there are a number of stimuli rendered in the previously outcome-associated color in a dense array and observers are free to report either of two color-defined targets, such that involuntary attentional capture by stimuli of either color is unlikely to account for these patterns of results (see also Clement & Anderson, 2023); it instead appears the selection history is influencing which color stimuli an observer chooses to search through. The idea that these observed biases are uniquely consistent with an effect of selection history on voluntary, goal-directed attention is further attested to by the fact that reward and punishment have opposite effects on the attentional control settings employed in the service of finding a target (at least for individuals not high in anxiety), with punishment-related stimuli being deprioritized; this contrasts with involuntary attentional biases for stimuli previously associated with reward and punishment, which in both cases involve elevated priority for the outcome-associated stimulus through a common underlying mechanism tied to survival relevance (e.g., H. Kim & Anderson, 2023; H. Kim et al., 2021a, 2021b). In summary, we see evidence that reward and punishment history shape how a person chooses to search for a target in a manner that is distinct from involuntary attentional biases for outcome-associated stimuli, biasing this choice in a manner that runs counter to what would be the most optimal for performance maximization.

Consistent with the findings of Lee et al. (2025), Britton and Anderson (2021) measured eye movements in a task in which observers needed to fixate colored stimuli in order to reveal a hidden target, with stimuli rendered in each of several colors equally likely to constitute the target on a given trial. Importantly, fixating stimuli rendered in one particular color sometimes triggered an aversive electric shock. Under these conditions, observers exhibited a tendency to avoid fixating stimuli rendered in the shock-associated color, effectively ignoring these stimuli until other potential targets had been exhausted (Britton & Anderson, 2021). As with Lee et al. (2025), this bias contrasts with the involuntary influence of punishment history on attention, which produces a bias to orient toward signals for threat (Anderson & Britton, 2020, Anderson et al., 2022). Interestingly, there was some evidence for an initial covert attentional bias toward the shock-associated stimuli in Britton and Anderson (2021) that was then overridden by goal-directed attentional processes influencing the control of overt attention, with the initial involuntary covert attentional bias potentially serving as a means of marking a stimulus for suppression at later stages of oculomotor selection (e.g., Moher & Egeth, 2012).

Eye movements during naturalistic viewing have additionally been shown to be biased by reward learning and aversive conditioning. When observers are foraging for a target, the direction of their initial eye movement is biased in the direction of orienting that tended to yield high reward in a previous spatial selection task (Liao & Anderson, 2020b). Unlike spatial attentional biases evident in a subsequent target detection task (Anderson & Kim, 2018a, 2018b; Chelazzi et al., 2014; Liao et al., 2023; Mine et al., 2021), in Liao and Anderson (2020b), participants were presented with a free choice concerning the direction in which to begin searching and displays were visually balanced, requiring that equipotent stimuli be serially fixated until a hidden target was revealed; it therefore stands to reason that reward history was impacting the manner in which observers executed an endogenously generated saccade. In the context of aversive conditioning, delivering an electric shock contingent on the execution of a saccade of a particular direction and amplitude was found to reduce the tendency to generate saccades of that direction and amplitude during free viewing of pictures for a later memory test (Anderson, 2021c). The direction of the bias was opposite the involuntary effect of aversive conditioning on spatial attention, which results in elevated priority afforded to locations at which selection has previously yielded an aversive outcome, likely reflecting the involuntary facilitation of threat monitoring (Anderson et al., 2022). The bias observed in Anderson (2021c) also occurred in the absence of awareness concerning the relationship between electric shocks and eye movements.

Collectively, the above-reviewed studies demonstrate an effect of reward and punishment history on the choice of how to allocate attention across features and space. This effect can be observed in extinction (Anderson, 2021c; Britton & Anderson, 2021; Lee et al., 2022, 2025; Liao & Anderson, 2020b), when the goals of the task and/or the task itself has changed (Anderson, 2021c; Britton & Anderson, 2021; Lee et al., 2022, 2025; Liao & Anderson, 2020b), when the bias runs counter to a more efficient search strategy (Lee et al., 2022, 2025), and when observers are unaware of the contingencies responsible for the bias (Anderson, 2021c). That is, we see evidence for an influence of selection history on the voluntary control of attention that exhibits the characteristics of habit-like attention that include inflexibility and the ability to proceed in the absence of awareness. The above-described influence of punishment history on attentional biases also runs counter to the nature of involuntary punishment-related attentional biases driven by selection history (Anderson, 2021c; Britton & Anderson, 2021; Lee et al., 2025), further consistent with unique influence on the voluntary, goal-directed control of attention.

Additional evidence suggests that the influence of reward and punishment history on voluntary, goal-directed information processing is not limited to the control of attention. Attending to a stimulus and encoding a stimulus into memory can be decoupled, such that attending to a stimulus does not necessarily result in its attributes being encoded into working memory (e.g., Chen & Wyble, 2015, 2016; Chen et al., 2016; Eitam et al., 2013, 2015; Fu et al., 2023; R. Wang et al., 2021). The failure to encode an attribute of an attended stimulus into working memory can even occur for the attribute that distinguishes the target from non-targets (e.g., Chen & Wyble, 2015, 2016; Chen, Yan et al., 2019a, 2019b; Chen, Yu et al., 2019a, 2019b), provided that the reported attribute of the target is different from its defining attribute used to guide search (e.g., Botella et al., 2001; Remington & Folk, 2001). In this sense, it can be argued that working memory encoding is guided by a template for the stimulus attributes or features needed to inform a behavioral response, much like attention is guided by a mental representation of the target of search. When an object possesses a feature/attribute that was previously associated with reward or punishment, such as a particular color, it was recently shown that the presence of this feature impairs the encoding of other features of the object (Yan et al., 2024). This suggests that selection history impacts not only the strategic control of attention, but also the intentional encoding of information into working memory, influencing the selectivity of goal-directed information processing more broadly.

### Experience-dependent tuning of attentional control

Beyond reward and punishment history, the act of repeatedly selecting a stimulus in the absence of explicit reward and punishment outcomes is alone capable of generating a persistent attentional bias that potentiates the future selection of the stimulus (e.g., Anderson & Britton, 2019; H. Kim & Anderson, 2019b; Kyllingsbaek et al., 2001; Sha & Jiang, 2016; Shiffrin & Schneider, 1977). As with reward- and punishment-related effects, some of this history-dependent influence is clearly involuntary, capable of competing with attention to an instructed target (e.g., Anderson & Britton, 2019; H. Kim & Anderson, 2019b; Sha & Jiang, 2016). However, recent research points to a more direct influence on how a person chooses to allocate attention when given a choice of how to search as well, suggesting an influence of prior selection on the content and implementation of voluntarily instantiated attentional control settings.

Early evidence for an influence of past attentional selection on attentional control settings was provided by the effect of selection history on search modes. Leber and Egeth (2006a, 2006b) conducted experiments in which participants were forced to identify a target on the basis of its contrast in the shape dimension (i.e., *singleton detection mode*) or on the basis of its specific shape identity (i.e., *feature search mode*). All participants were then presented with “option” trials in which it was possible to identify the target either on the basis of its specific shape or contrast in the shape dimension. Across three studies (Leber & Egeth, 2006a, 2006b; Leber et al., 2009), it was found that the manner in which participants had learned to search when forced to adopt a particular search mode predicted how they would later search on these option trials, suggesting that they had learned to adopt a particular attentional control setting from past search experience. Cosman and Vecera (2013) further demonstrated that this selection history-dependent effect can arise in a context-dependent manner, with self-reported strategies suggesting the absence of explicit context-dependent set shifting. Such biases can be observed in spite of the fact that one of the two search modes—namely, feature search mode—has been shown to be less effortful and generally preferred over singleton detection mode when participants are given an explicit choice of search mode (Lee et al., 2024).

Using the ACVS task, A. J. Kim et al. (2024) demonstrated that participants were more likely to search using the optimal strategy when that strategy had been more advantageous during a prior training phase. Prior experience with search conditions under which it was particularly advantageous to identify and search through a less numerous set of colored stimuli resulted in participants developing a tendency to persist in the use of this strategy, even though this strategy was less advantageous than it had been previously. Lee et al. (2023) provided evidence for a parallel effect of selection history promoting a suboptimal strategy. When the target of search was more likely to be rendered in a particular color during an initial training phase, participants were biased to choose to search among stimuli of this color even when it was disadvantageous to do so. Together, A. J. Kim et al. (2024) and Lee et al. (2023) demonstrate that the visual search strategy that a person adopts is to some degree influenced by which strategy has proven effective in the past, which can both facilitate and hinder efficient search depending on the fit between that strategy and current search conditions.

In addition to the persistence of prior attentional control settings and strategy, how an individual chooses to search is also influenced by the statistics of the task. When a particular color target is likely to appear in the more advantageous (i.e., less numerous) set of colored stimuli to search through, participants develop a tendency to choose to search through stimuli of that color that persists into epochs in which it is no longer advantageous to search in this manner (Clement & Anderson, 2023). Importantly, this bias occurred in the absence of evidence that participants involuntarily attended to stimuli rendered in the color they were biased to choose to search through, as no distractor costs were observed for task-irrelevant stimuli rendered in this same color (Clement & Anderson, 2023). The observed bias was also evident for participants who were unaware of color contingencies responsible for it.

A recent body of research suggests that the content of attentional control settings, and specifically the mental template used to bias attention in favor of a searched-for target (i.e., the *target template*), operates via a “good enough” principle that is shaped by prior learning (Yu et al., 2023). For example, observers may learn to first search for a conspicuous object frequently associated with the location of the searched-for target in order to anchor their search to a high-probability location (Boettcher et al., 2018; Võ et al., 2019). When a target object is reliably paired with a non-target object in a particular spatial arrangement, the paired non-target object will be incorporated into attentional control settings (Clement & Anderson, 2024). Non-diagnostic features of a target will also become incorporated into attentional control settings when previous instances of the target possess these features and they provided useful information for distinguishing the target from other objects in the environment (Addleman et al., 2024; Bahle et al., 2021; see also Zhang & Carlisle, 2023). It seems that observers do not search for what task instructions emphasize or even for the target object itself, but rather on the basis of what has proven useful for localizing the target in the past.

Inter-trial priming, where a target feature that repeats over successive trials facilitates search and a distractor feature that then becomes a target feature on the following trial impairs search (see Geyer et al., 2006; Kristjánsson, 2006; Kristjánsson & Campana, 2010; Kristjánsson & Driver, 2008; Kristjánsson et al., 2002; Lamy et al., 2008; Maljkovic & Nakayama, 1994, 1996), could in some respects be described as an effect of selection history on the goal-directed control of attention. Ramgir and Lamy (2022) systematically argue that the effects of inter-trial priming are more robust when the task requires or encourages participants to maintain the primed feature in working memory or form expectations about the upcoming target, suggesting that the volitionally activated representation of the primed feature and its effects on the control of attention are modulated by recent experience (see also Wolfe et al., 2003). This is not to imply that inter-trial priming does not also encompass involuntary modulations of attentional priority, which are reflected in contemporary accounts of selection history (Anderson et al., 2021; Anderson, 2024b). Indeed, feature-priming effects remain robust when the target color is entirely predictable (e.g., Gaspelin et al., 2019; Maljkovic & Nakayama, 1994), as does location-priming (as measured via eye movements) when the target location never repeats and participants are informed of this (Talcott & Gaspelin, 2020). However, such involuntary modulations of attentional priority do not provide a complete account of the nature of inter-trial priming.

Beyond the content of attentional control settings, the influence of selection history on the goal-directed or strategic control of attention can also be seen in the context of motivation effects. It is well documented that the precision and efficiency of goal-directed attentional control is sharpened by the motivation provided by reward incentive (e.g., Esterman et al., 2017; Etzel et al., 2016; Padmala & Pessoa, 2011; Pessoa & Engelmann, 2010; Small et al., 2005). The effects of motivation on goal-directed information processing have both sustained and transient components (e.g., Braver, 2012; Jimura et al., 2010), and recent evidence demonstrated that feedback-related motivation to resist overtly attending to a task-irrelevant distractor has a persistent effect on attentional control that can be observed well after performance feedback has been removed (Anderson & Mrkonja, 2021, 2022). Such evidence suggests that attentional control settings adjusted in response to the motivational impact of performance feedback, and this motivation-related adjustment was incorporated into the attentional control settings participants came to associate with the performance of the task, perhaps with analogy to the persistent impact of enforcing the use of a particular search mode (Leber & Egeth, 2006a, 2006b). That is, how the observer searched in the past predicted how they searched in the future, in a manner that is somewhat robust to changes in the circumstances of the task (assuming that the past manner of searching is still tenable).

Collectively, the above-reviewed studies demonstrate an effect of past episodes of attentional selection and statistical learning on the choice of how to allocate attention across features and space. Like the effects of reward and punishment described in the preceding section, these effects can be observed when the contingencies responsible for their learning are no longer in place (Clement & Anderson, 2023, 2024; Cosman & Vecera, 2013; Leber & Egeth, 2006a, 2006b; Leber et al., 2009; Lee et al., 2023), when the goals of the task and/or the task itself has changed (Cosman & Vecera, 2013; A. J. Kim et al., 2024; Leber & Egeth, 2006a, 2006b; Leber et al., 2009; Lee et al., 2023), when the bias runs counter to a more efficient search strategy (Leber & Egeth, 2006a, 2006b; Leber et al., 2009; Lee et al., 2023), and when observers are unaware of the contingencies responsible for the bias (Clement & Anderson, 2023; Cosman & Vecera, 2013). Once again, we see evidence for an influence of selection history on the voluntary control of attention that exhibits the characteristics of habit-like attention.

As with the influence of reward and punishment, the influence of past experience in the absence of explicit outcomes extends beyond the voluntary control of attention and can also bias the manner in which information is encoded into working memory. We might refer to the operative nature of past experience as *encoding history* in this context. When a particular stimulus attribute needs to be reported for one type of target (e.g., a letter), participants will come to encode the same attribute of other targets (e.g., digits) that generally do not require the reporting of that attribute (Yan et al., 2023). Conversely, even when participants have experience reporting a particular attribute of a target, they exhibit a tendency to no longer encode this attribute when they have experienced a series of trials in which that attribute did not need to be reported (Yan & Anderson, 2024; see also Zivony & Eimer, 2022). Again, we see evidence that the influence of past experience or selection history is not limited to goal-directed attentional processes and instead reflects a broader principle influencing goal-directed information processing across cognitive systems.

## Potential underlying mechanism

Anderson (2024b) argued that the control of attention is fundamentally memory-dependent, with the effect of current task goals and selection history contributing to activated memory representations that collectively constitute the attentional control state and function to bias information processing in sensory systems. This conceptual framework provides a straightforward mechanism by which selection history could influence voluntary, goal-directed attention in addition to exerting a more direct influence. Specifically, it is possible that selection history results in the development of relatively stable and inflexible context-specific memories that, when later activated in similar contexts, facilitate the activation of corresponding representations in more flexible memory systems that serve in the interest of goal-directed attention (see Ballard et al., 2024). In essence, selection history could bias competition in flexible memory systems that serve in the interest of guiding attention (see van Ede & Nobre, 2023), resulting in particular attentional templates being more quickly and readily recruited under familiar task conditions. That is, a particular attentional template or search strategy develops a tendency to become “top of mind” on the basis of prior experiences in similar task contexts. There might further be a cyclical effect in which selection history shapes the manner in which task-related information is encoded (see Yan & Anderson, 2024; Yan et al., 2023), which in turn shapes the information that is represented and available to guide the choice of attentional control settings. In these ways, observers may develop “knee-jerk” ways of intentionally processing displays and become myopically “locked in” to these modes of processing, provided that they remain functional or “good enough” (see Yu et al., 2023) when it comes to facilitating the attainment of task goals.

The idea of selection history influencing the goal-directed or strategic control of attention is also consistent with an adaptive view of selection history-dependent attentional control in which this attention mechanism serves, in part, to offload the need for controlled and effortful information processing (Anderson, 2021a). Visual search can in many cases be characterized as an effortful cognitive process (Anderson & Lee, 2023; Anderson et al., 2025). By offloading the strategic control of attention to habit-like influences, the need to actively monitor the specific conditions of the task and decide on an appropriate attentional control setting to instantiate is substantially reduced, perhaps reserved predominantly for unfamiliar situations or recruited in response to situations in which task performance falls below a desired threshold. Anderson (2021a) argued for the adaptive nature of the influence of selection history on the involuntary control of attention, but the same arguments concerning benefit amplification and cost minimization can be applied to the effects of selection history on the voluntary, goal-directed control of attention.

Our linking of selection history to the goal-directed control of attention is consistent with models of attentional control that broadly define a control state that collectively encompasses these influences while maintaining a mechanistic distinction between them (e.g., Anderson, 2024b; Luck et al., 2021; Todd & Manaligod, 2018). We would hypothesize a stronger connection between selection history and goal-directed influences than these models might be taken to imply. Such a connection may contribute to the hypothesized relationship between signal suppression and selection history (Gaspelin et al., 2025), with the past rejection of a distractor strengthening the effectiveness with which that stimulus can be rejected as task-irrelevant in the future. Whereas prior models of attentional control have emphasized an influence of goal-directed attention on selection history (e.g., Anderson et al., 2021; Wolfe, 2021), we hypothesize that this relationship is bidirectional.

While we argue that selection history plays an important role in determining which attentional control settings observers choose to adopt, these control settings are by no means solely reducible to selection history-dependent influences, even in cases where past modes of attentional selection remain effective under current task conditions. Observers are able to choose to search in ways that are not consistent with their selection history and indeed exhibit a broad range of search strategies across trials even in the presence of robust selection history (e.g., A. J. Kim et al., 2024; Lee & Anderson, 2023; Lee et al. 2022). With that said, voluntary, goal-directed attentional control is not the product of a blank slate that the intentions and motivations evoked by the current task context write upon. It rather seems to be the case that selection history biases the content of attentional control settings toward what has proven effective in the past, elevating the strength with which alternative control states would need to be activated in order to influence attention otherwise.

## Theoretical implications

As we have argued throughout this paper, goal-directed attentional processes are not insulated from experience-dependent influences in their instantiation or implementation. The studies discussed in this review amplify and provide additional theoretical context for the assertion made by Anderson (2018) that goal-directed attentional control may in some cases reflect experience-dependent priority weighting. Anderson (2018) argued this point in the context of involuntary effects of selection history on attentional control supporting the attainment of task goals. Here, we claim that goal-directed attentional processes are themselves subject to habit-like influences, shaped not only by current intentions but also by past experience. It is presumptive to assume that what a person chooses to search for and how they choose to proceed with their search are limited to the product of decisions that follow directly from instruction and current task demand; a full understanding of goal-directed attention requires an understanding of the role of selection history in task-related information processing.

At the same time, selection history effects on attention are not restricted to the domain of involuntary attentional control and attentional capture. Although evidence strongly points to an involuntary influence of selection history on the control of attention that is dissociable from voluntary, goal-directed mechanisms of control (e.g., Anderson, 2013; Anderson & Britton, 2020; Anderson et al., 2011; Le Pelley et al., 2015), the involuntary aspects of selection history reflect just one dimension of selection history-dependent attentional control. There exists another dimension of selection history, one that functions to shape the representations that are volitionally brought to bear in the pursuit of a task goal. This influence of past experience on the nature of goal-directed attentional control needs to be considered whenever volitional attentional processes are measured.

More broadly, the findings reviewed in this paper argue for a fuzzy boundary between goal-directed and selection history-dependent influences on the control of attention. As argued by Anderson (2024b), although goal-directed and selection history-dependent mechanisms of attentional control are clearly distinguishable, this distinction is not a categorical one. Attentional control settings are complex and multifaceted, with contributions from representations that are volitionally brought online in the service of momentary task goals and representations that are stored in long-term memory and activated by the current task context. It would be perhaps surprising if these diverse components of the attentional control setting did not to some degree influence each other in the process of coordinating attentional selection.

This is also not to imply that the influence of selection history on the voluntary control of attention is restricted to habit-like influences. Indeed, to the degree that observers become aware of the contingencies responsible for selection history-dependent effects, they may choose to update their attentional control settings to better take advantage of these contingencies (see Addleman & Jiang, 2019). In this case, selection history is updating the knowledge that observers intentionally draw upon when configuring strategic attentional control. In addition to this more indirect knowledge-based influence, we argue that selection history also produces learning that can exert a more direct, habit-like influence on the choice of attentional control settings, biasing how observers choose to search in a manner that does not depend on explicit knowledge (e.g., Clement & Anderson, 2023; Lee & Anderson, 2023). The distinction here maps onto the concept of experience-*dependent* influences (which can include indirect influences of selection history via explicit knowledge) versus experience-*driven* influences (which are limited to direct influences of selection history) on the control of attention as articulated by Anderson et al. (2021).

**Fig. 1:**
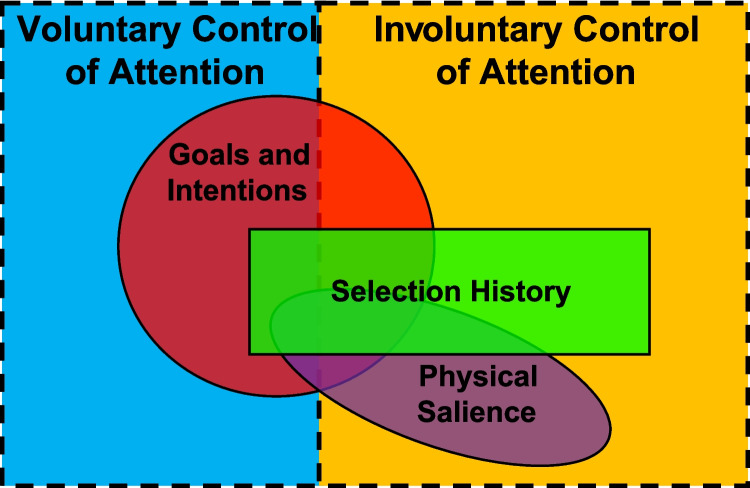
Proposed model relating the influence goals and intentions, selection history, and physical salience to the voluntary and involuntary control of attention

We suggest a model of attentional control that reflects the relationship between selection history and the goal-directed control of attention argued in this review (Fig. 1). The influence of selection history spans both the involuntary and voluntary control of attention. On the side of involuntary control, selection history can have a unique influence that is independent of the influence of both goals and intentions as well as physical salience (reflected in the area of non-overlap on the involuntary side of Fig. 1), while on the side of voluntary control, the influence of selection history is entirely subsumed within its relationship to goal-dependent influences.

It is worth emphasizing that the proposed relationship between selection history and the voluntary control of attention is complex and, in some respects, defies a straightforward distinction between voluntary and involuntary processes. We have argued that, in addition to inputs traditionally associated with voluntary attention (e.g., motivation, task goals), selection history also influences the choice of attentional control settings. In this respect, the voluntary control of attention is shaped by selection history. At the same time, the proposed mechanism(s) by which selection history bias this choice operate in a habit-like fashion that can proceed in the absence of explicit volition, which is a feature of involuntary attentional control. As such, it can be argued that what we are proposing is an expanded perspective on the nature of involuntary attentional control, extending involuntary influences into the domain of the strategic control of attention. While it seems clear that voluntary attentional control processes are subject to selection history-dependent influences, *how* these influences operate mechanistically and whether this influence is itself best characterized as voluntary or involuntary in nature are important questions for future research, both empirically and philosophically (e.g., Wu, 2023). What is reflected in Fig. 1 is our hypothesis that the attentional control settings a person chooses to adopt when performing a task can be subject to selection history-dependent influences, in some cases reflecting the joint product of selection history and goals and intentions (hence the overlap between the two on the side of voluntary control). Likewise, the goals and intentions that are volitionally brought to bear in the control of attention are subject to selection history-dependent influences; it is not the case that the influence of selection history is restricted to a direct influence on attentional priority that operates outside of the purview of goals and intentions.

Although the focus of this review has been on the relationship between selection history and the voluntary, goal-directed control of attention, the broader complexion of the model (see Fig. 1) bears explanation. The role of goals and intentions in the domain of the involuntary control of attention reflects contingent attentional capture, in which stimuli possessing a task-relevant feature are prioritized even when they are known to themselves be task-irrelevant (e.g., Folk et al., 1992; Luck et al., 2021); while the configuring of the attentional control state is to some degree voluntary in the case of contingent attentional capture, the implementation of the control state can be involuntary. The influence of selection history on the configuration of the attentional control state in the context of contingent attentional capture (e.g., Cosman & Vecera, 2014; Luck et al., 2021) and of the modulatory influence of the volitional components of the control state on involuntary selection history-dependent influences (Anderson, 2024b) is reflected in the overlap between selection history and goals and intentions on the involuntary side of control depicted in Fig. 1. The overlap between physical salience and goals and intentions on the voluntary side of control reflects the engagement of search modes that prioritize or otherwise strategically allow for salient stimuli to strongly influence the allocation of attention (e.g., Bacon & Egeth, 1994; Lamy & Egeth, 2003; Leber & Egeth, 2006a, 2006b), and on the involuntary side reflects the modulatory role of salience in contingent attentional capture and the modulatory role of task goals in stimulus-driven attentional capture (e.g., Eimer & Kiss, 2008; Eimer et al., 2009; Gaspelin et al., 2016; Lu & Han, 2009). The idea that attentional control settings as subsumed within search modes can strongly modulate the influence of salience on the computation of attentional priority has received strong support from the signal suppression hypothesis (e.g., Chang & Egeth, 2019; Gaspelin et al., 2015, 2017). All of these relationships are likely subject to selection history-dependent modulation (Anderson et al., 2021; Anderson, 2024b), hence the three-way overlap. As an extension of arguments made by Anderson (2018, 2021a, 2024a, 2024b; see also Theeuwes, 2018), the domain of involuntary attentional control is more expansive than the domain of voluntary attentional control, owing to the claim that the allocation of attention generally proceeds in a manner that is outside of the observer’s control by default. Note that areas of overlap depicted in Fig. 1 reflect situations in which two or more factors can jointly influence attentional priority computations and do not necessarily imply an interaction between these influences.

## Future directions

In supporting our hypothesis that selection history influences the voluntary, goal-directed control of attention, we reference studies spanning the different components of selection history as outlined by Anderson et al. (2021): association with valent outcomes (e.g., reward: Lee et al., 2022; punishment: Lee et al., 2025), target history (e.g., Lee et al., 2023), statistical learning (e.g., Clement & Anderson, 2023), and inter-trial priming (e.g., Ramgir & Lamy, 2022). Each of these sources of prior experience influences the involuntary control of attention in distinct ways (see Anderson et al., 2021), and there is good reason to suspect corresponding diversity to the mechanisms underlying the influence that such learning has on the voluntary control of attention. Such diversity may be related to the different memory systems implicated in the learning (see Anderson, 2024b) and their connections with the flexible memory systems supporting the voluntary, goal-directed control of attention. Future research could more systematically compare and contrast the degree of influence that different components of selection history have on the voluntary, goal-directed control of attention, which would have the potential to provide unique insights concerning the architecture of the attention system.

In light of the arguments forwarded in this review, it is worth emphasizing that selection history-dependent influences on the control of attention have historically been characterized as, by definition, involuntary and capable of competing with goal-directed attentional processes (see Anderson et al., 2021). All of the influences of selection history on the voluntary, goal-directed control of attention explored in this review were identified and theoretically developed in the context of their link to the involuntary control of attention. If selection history can exert a habit-like influence on the voluntary control of attention, as hypothesized in this review, this scientific history places an arbitrary constraint on the learning-dependent processes examined. Habit-like influences on the voluntary control of attention need not have an involuntary counterpart, and there may be other habit-like influences on these processes beyond the traditionally accepted components of selection history. The habit-like influences of associations with valent outcomes, target history, and other forms of prior experience discussed in this review may therefore reflect a subset of the learning that can have the hypothesized influence on the control of attention, which is a possibility ripe for scientific exploration.

How do we know that an effect of selection history on the control of attention is specifically modulating a voluntary attentional process, especially given the well-documented influences of selection history on the involuntary control of attention (Anderson et al., 2021)? Anderson (2018) outlined how seemingly goal-directed attentional selection may in fact be supported by involuntary processes that happen to align with current task goals, which creates unique challenges when it comes to isolating an influence of selection history on the voluntary control of attention: Just because an effect of selection history is compatible with the goals of the task does not mean that it must reflect a voluntary process. The converse, isolating an involuntary influence of selection history, is much more straightforward given that situations can be created in which a putative influence of selection history is in opposition to an influence of task goals (e.g., Anderson et al., 2011; Gaspelin et al., 2019). For research on the influence of selection history on the voluntary control of attention to progress, a robust framework for confidently isolating this influence is needed.

Although the development of such a framework will undoubtedly benefit from refinement in the context of future research, we would note two potentially promising avenues. The first, adopted by several of the reviewed studies, is to infer voluntarily instantiated attentional control states from the targets that participants report finding when given the choice of two or more targets for which to search (Clement & Anderson, 2023; A. J. Kim et al., 2024; Lee & Anderson, 2024a, 2024b; Lee et al., 2022, 2025; see also Britton & Anderson, 2021; Liao and Anderson (2020b). Compelling evidence in favor of the validity of this approach is provided by findings in which the observed influence of selection history runs counter to the kind of influence that would be expected from an involuntary influence (e.g., Anderson, 2021c; Anderson et al., 2022; Lee et al., 2025). The second, which could be adopted in concert with the first, is to contrast performance under conditions in which involuntary orienting biases are assessed by pitting task goals against attention to the stimulus that was putatively afforded higher priority via mechanisms of voluntary attentional control (e.g., Clement & Anderson, 2023) or under conditions in which participants are given explicit instruction to search otherwise. If no evidence of involuntary orienting to a stimulus that was preferentially attended when task-relevant as a function of selection history is observed under similar task conditions or if this bias vanishes when participants are instructed to search differently, the argument that the bias was evident in the voluntary condition of attention stands on firmer ground.

## Conclusions

Theories concerning the influence of selection history on the control of attention have focused almost exclusively on involuntary influences on the computation of attentional priority that are distinct from, and can compete with, goal-directed influences (e.g., Anderson, 2016; Anderson et al., 2021). Here, we argue that the role of selection history in the control of attention is not limited to such involuntary adjustments in the attentional priority of stimuli. Rather, the voluntary, goal-directed control of attention is itself subject to habit-like influences of selection history, reflecting a robust role for prior experience in addition to the volitional impact of momentary goal-related priorities and intentions. A complete understanding of voluntary, goal-directed attentional control requires an understanding of selection history, and a complete understanding of selection history cannot be divorced from volitional attentional processes. The idea of “attention on autopilot” (Leber & Egeth, 2006a) is a robust concept in the domain of attentional control, with significant explanatory power that is worthy of further exploration and should be incorporated into any comprehensive theory of the mechanisms underlying the control of attention.

## Data Availability

Not applicable.
